# Long-term outcomes of autologous platelet treatment for optic disc pit maculopathy

**DOI:** 10.1007/s00417-023-06159-1

**Published:** 2023-07-04

**Authors:** Konstantinos Gklavas, Alexandros Athanasiou, Jonas Neubauer, Evangelia Lilou, Lisa Pohl, Karl Ulrich Bartz-Schmidt, Spyridon Dimopoulos

**Affiliations:** 1 Centre for Ophthalmology, Eberhard-Karls University, Tübingen, Germany; 2 Institute for Ophthalmic Research, Eberhard-Karls University, Tübingen, Germany

**Keywords:** Optic disc pit, Maculopathy, Autologous platelet concentrate, Macular detachment, Pars plana vitrectomy

## Abstract

**Purpose:**

Optic disc pits (ODPs) are rare congenital cavitary abnormalities of the optic nerve head, which can lead to serous macular detachments. The aim of this study was to evaluate the long-term efficacy of pars plana vitrectomy (PPV) combined with autologous platelet concentrate (APC) for the treatment of optic disc pit maculopathy (ODP-M).

**Methods:**

A retrospective analysis was performed on eleven eyes of ten patients with ODP-M, who received PPV combined with APC. Nine eyes operated primary, four of which had a repeat surgery also with injection of APC and two eyes underwent a rescue surgery, after they have been operated in another eye center without APC. Morphological and functional results were the main outcome parameters, determined by optical coherence tomography (OCT) and best-corrected visual acuity (BCVA), respectively.

**Results:**

The mean duration of visual loss before surgery was 4.7 ± 3.89 months (range 0–12 months). The mean BCVA increased significantly from 0.82 ± 0.33 logMAR (range 0.4–1.3) preoperatively to 0.51 ± 0.36 logMAR (range 0–1.2) at the last examination (*p* = 0.0022). A significant morphological improvement was also noticed with decrease of the mean foveal thickness from 935.82 ± 248.48 µm (range 559–1400 µm) preoperatively to 226.45 ± 76.09 µm (range 110–344 µm) at the final examination (*p* < 0.0001). The patients were followed-up for a mean 65.36 ± 48.81 months (range 1–144 months). Two eyes developed postoperatively a retinal detachment. Cataract surgery was performed in 5 eyes during the follow-up period.

**Conclusion:**

Our study demonstrated that PPV with APC can improve functional and morphological outcomes, both as a primary and a rescue therapy, without any recurrence over a long follow-up period. To the best of our knowledge, this was the longest observation period regarding the use of APC in treatment of ODP-M.

**Supplementary Information:**

The online version contains supplementary material available at 10.1007/s00417-023-06159-1.


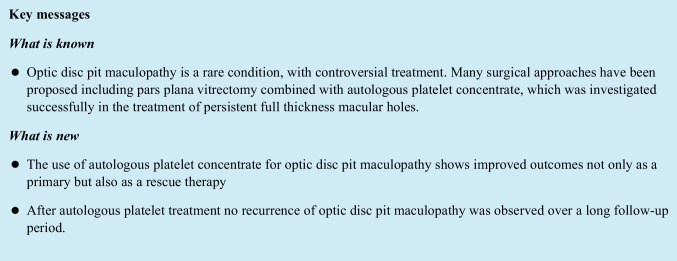


## Introduction

**Fig. 1:**
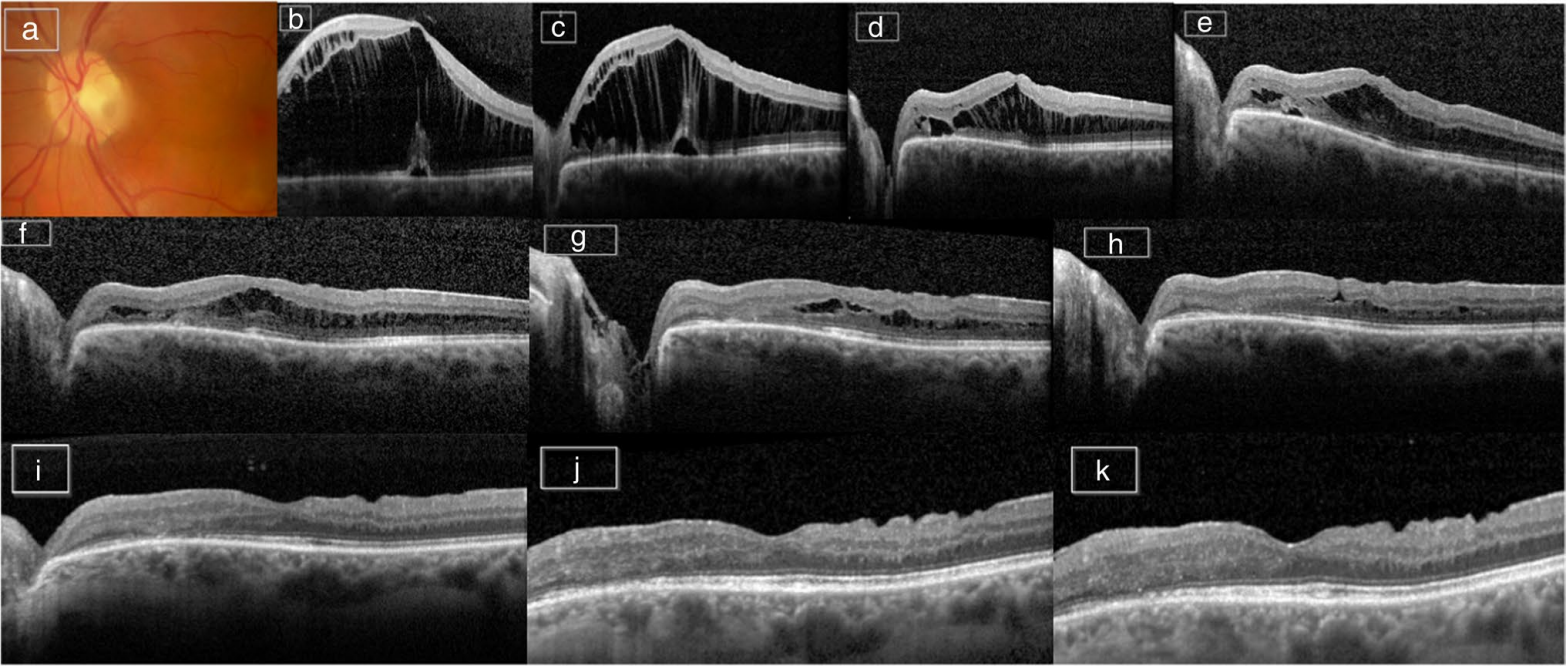
Case 1: this patient presented with ODP-M and underwent PPV + ILM peeling with APC injection and because of a persistent ODP-M underwent repeat PPV with APC injection after two months. **a** Preoperatively color fundus photography of optic disc. **b** OCT one day before first surgery. **c** OCT 2 months after first surgery and one day before second surgery. **d** OCT 2 months after second surgery. **e** OCT 4 months after second surgery. **f** OCT 7 months after second surgery. **g** OCT 13 months after second surgery. **h** OCT 19 months after second surgery. surgery. **i** OCT 73 months after second surgery. **j** OCT 113 months after second surgery. **k** OCT 144 months after second surgery

**Fig. 2:**
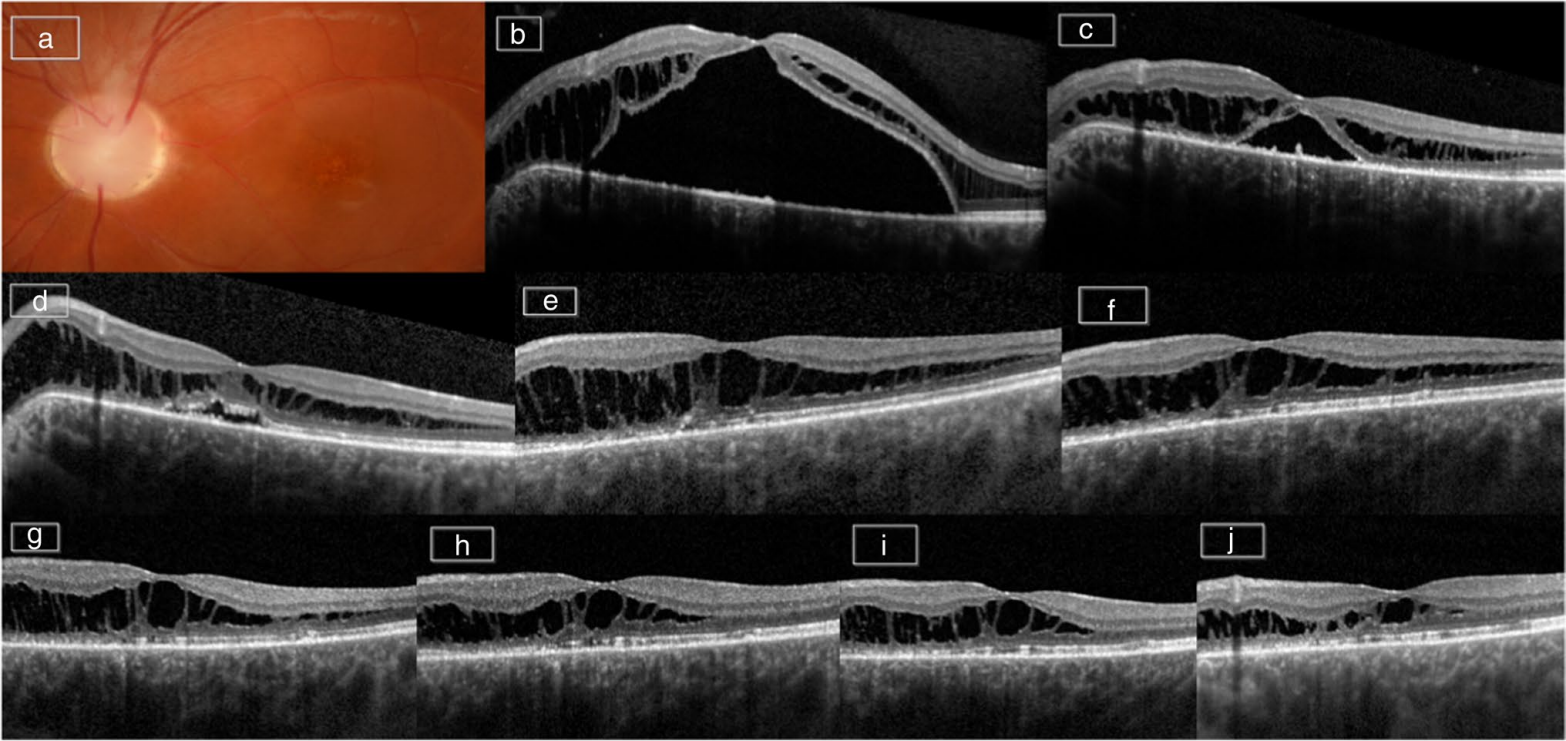
Case 7 left eye: this patient presented with ODP-M and underwent PPV with APC injection. **a** Preoperatively color fundus photography of optic disc. **b** OCT one day before surgery. **c** OCT 2 weeks postoperatively. **d** OCT 4 months postoperatively. **e** OCT 13 months postoperatively. **f** OCT 19 months postoperatively. **g** OCT 25 months postoperatively. **h** OCT 44 months postoperatively. **i** OCT 50 months postoperatively. **j** OCT 59 months postoperatively

**Fig. 3:**
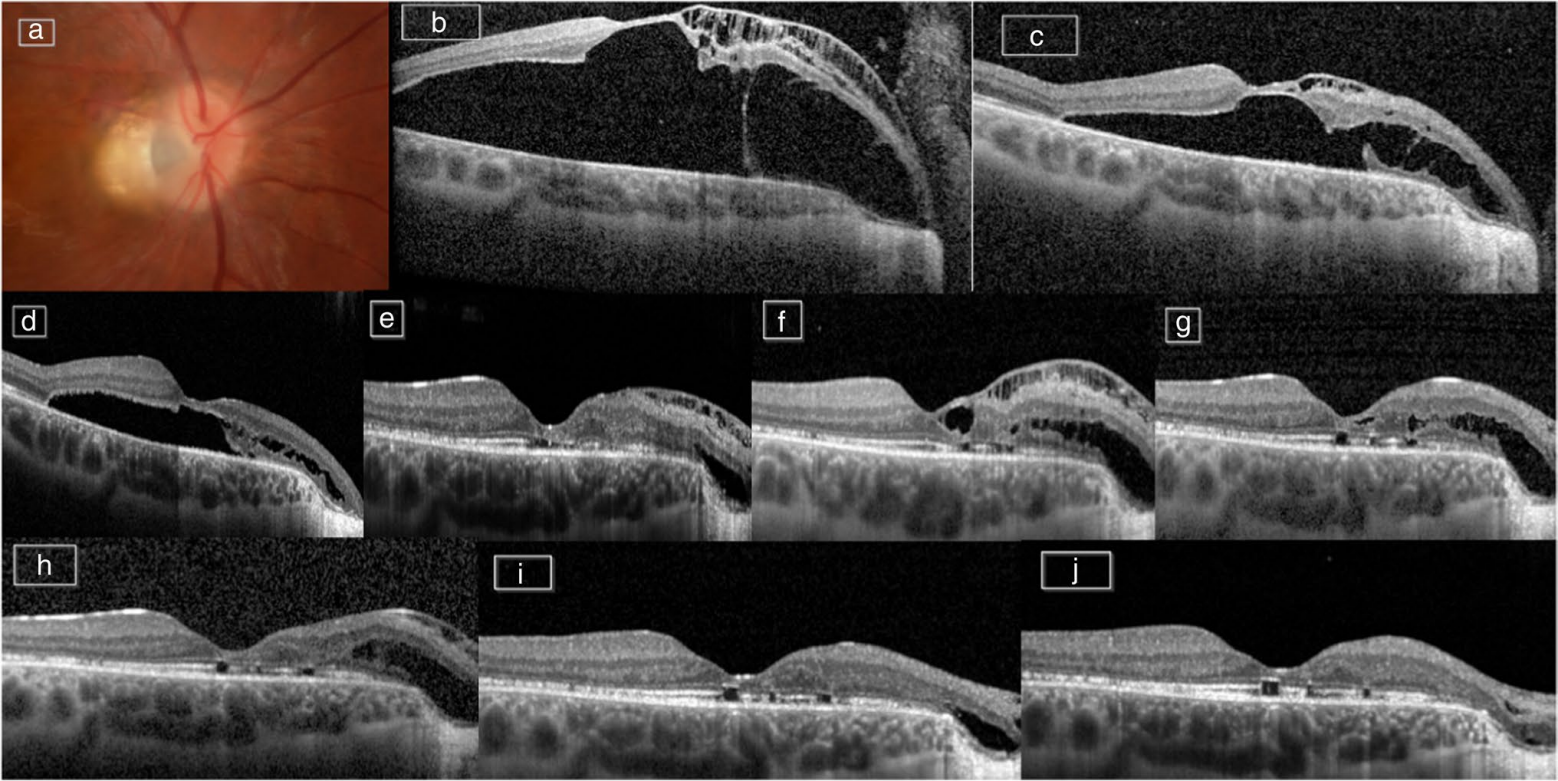
Case 10: this patient presented with ODP-M and underwent PPV with APC injection. **a** Preoperatively color fundus photography of the optic disc. **b** OCT one day before surgery. **c** OCT 42 days postoperatively. **d** OCT 5 months postoperatively. **e** OCT 12 months postoperatively. **f** OCT 24 months postoperatively. **g** OCT 30 months postoperatively. **h** OCT 36 months postoperatively. **i** OCT 42 months postoperatively. **j** OCT 48 months postoperatively

Optic disc pits (ODPs) are rare congenital cavitary abnormalities of the optic nerve head [1]. Both sexes are equally affected, they occur with an estimated prevalence of 1 in 11,000 ophthalmic patients and are unilateral in 85–90% of the cases [1, 2]. ODPs occur mostly sporadically but autosomal-dominant inheritance patterns have been observed [3, 4]. They are typically detected at the inferotemporal segment and appear as single round or oval, grayish depressions at the optic disc [5] (Figs. 1a, 2 and 3). Approximately half of the patients with ODP develop serous macular detachments and/or retinoschisis of the central macula, leading to the so-called optic disc pit maculopathy (ODP-M), which is most common in the third and fourth decades of life but may occur also during childhood or later in life [6–8]. There are several controversial suggestions about the origin of subretinal fluid such as cerebrospinal fluid, liquefied vitreous entering through the pit or through a macular hole, and leakage from either choroidal vessels or permeable vessels in the pit [5, 6, 8]. Various therapeutic approaches have been proposed such as laser coagulation of the retina, macula buckling, and pars plana vitrectomy (PPV) combined with further surgical steps [5, 9–14]. The goal of every approach is to eliminate the sub- and intraretinal fluid and also to seal the hypothetical communication between the vitreous cavity and the subretinal space as it is known, that subretinal fluid leads to a decrease of best-corrected visual acuity (BCVA) over time [15]. Autologous platelet concentrate (APC) as an adjuvant was investigated in the treatment of persistent full-thickness macular holes (FTMH) after ILM peeling, and a successful closure of the macular holes was achieved in 78% of cases [16, 17]. The aim of this study was to evaluate the efficacy of PPV combined with APC for the treatment of ODP-M.

## Patients and methods

A retrospective single-center interventional case study series was conducted. Only eyes diagnosed with ODP-M and which had been treated with PPV and APC were included in this study. The diagnosis of ODP-M was based on the typical clinical findings (excavation of the optic nerve head and elevation of the macula) and confirmed by optical coherence tomography (OCT), infrared, and color fundus photography in all eyes. Patients with a maculopathy other than ODP-M were excluded from the study. Exclusion criteria also included proliferative diabetic retinopathy, uncontrolled glaucoma, and rhegmatogenous retinal detachment in either eye. Surgery was offered only to patients with deterioration of BCVA due to progressive maculopathy. All patients had a complete eye examination, including BCVA, slit-lamp examination of the anterior segment, and fundus examination at baseline and at follow-up examinations. SD-OCT imaging (Spectralis HRA + OCT, Heidelberg Engineering GmbH, Heidelberg, Germany) was also performed preoperatively and in each follow-up examination, in order to monitor the status of the maculopathy. The nature of the disease and the possible consequences of the surgery were fully explained and documented in writing. All operations were performed by two surgeons at the University Eye Hospital, Tübingen, Germany, from June 2005 to July 2019.

Preoperatively, a hematological examination was performed, and any abnormalities of blood platelets were ruled out. Preparation of the autologous platelet concentrate was performed as described by Vote et al. [18]. One hour before the surgery, 16 ml of venous blood was drawn from the patients. The preparation of APC took place in the blood bank of University Hospital Tübingen. The blood sample was mixed with 4 ml of acid citrate dextrose formula A (ACD-A) and centrifuged at 280 g (1500 rpm) for 15 min. The platelet-rich plasma was then extracted and mixed with 1/8 volume of acid citrate dextrose and centrifuged again at 1500 g for 10 min. Finally, the platelet concentrate was mixed with 0.6 ml of sodium chloride. This suspension was then brought to the operating room within one hour of sampling and was collected in a sterile syringe using a sterile technique for intraocular use. Two drops of 0.1 ml each were then applied to the optic disc pit.

All patients underwent a 23-gauge three-port PPV. Posterior vitreous detachment (PVD) was induced by applying active aspiration with the vitreous cutter over the optic disc. ILM peeling was performed after staining with MembraneBlue-Dual™ (0.15% trypan blue, 0.025% brilliant blue G, and 4% PEG). After fluid-air-exchange APC was injected over the center of the pit. In all eyes, gas (perfluorethane-C2F6-16%) was used as endotamponade. All patients were instructed to maintain strict facedown position for at least 2 weeks. Four eyes of four patients, who already had undergone a PPV with injection of APC, two of them combined with internal limiting membrane (ILM) peeling, underwent a repeat surgery with injection of APC because of persistent ODP-M. Two eyes of two patients with persistent ODP-M, who have been treated in another eye center with PPV, underwent a rescue surgery with injection of APC in our clinic.

The central foveal thickness was measured, defined as the distance between the retinal pigment epithelium (RPE)/Bruch’s complex and the surface of the fovea. For the statistical analysis, the measurements obtained from the examination one day prior to surgery and from the last follow-up examination were used.

BCVA was measured preoperatively and in each follow-up examination using retroilluminated early treatment diabetic retinopathy study (ETDRS) charts (one line = five letters). For the statistical analysis, the measurements obtained prior to surgery and during the last follow-up examination were used. For purposes of statistical analysis, BCVA measured with ETDRS charts was converted to values corresponding to the logarithm of the minimum angle of resolution (logMAR).

The statistical analysis was carried out with JMP® 14 for Windows. Descriptive statistics for continuous variables expressed as mean, standard deviation (mean ± SD), and range. The results were analyzed with parametric statistical methods. The Shapiro–Wilk test was used to investigate the normality of the data. Changes of BCVA and foveal thickness between the baseline and last follow-up visit were calculated using the matched-pair *t*-test. The correlation between the surgical results and other parameters was evaluated using Pearson’s correlation coefficient *r*. A *p* value less than 0.05 was considered to be statistically significant.

## Results

In summary, eleven eyes of ten patients were analyzed in this study (two males/eight females). The mean age at the time of surgery was 32.36 ± 16.34 years (range 17–76 years, median 27 years). A bilateral ODP-M was detected in two patients. The first of them received surgery on both eyes, while the second one only on one eye (the fellow eye developed a spontaneous remission of the maculopathy). The pits were all located on the temporal side of the optic disc. Table 1 summarizes the intervention data, clinical characteristics, and surgical outcomes of the patients.

**Table 1:** Clinical characteristics, intervention data, and outcomes of study patients with optic disc pit maculopathy

Patients	Age	Gender	Eye	Duration of symptoms (months)	Preop BCVA logMAR (Snellen equivalent)	Foveal thickness preop (µm)	1. Operation	2. Operation	Postop BCVA logMAR (Snellen Equivalent)	Foveal thickness postop (µm)	Time to resolution of subretinal fluid (months)	Time to resolution of intraretinal fluid (months)	Follow-up (months)	Comments
1	39	M	L	6	0.7 (20/100)	1084	PPV + ILM peeling + APC	PPV + APC	0.5 (20/60)	250	13	25	144	Retinal detachment 1 week and cataract operation 21 months after 2^nd^ surgery
2	17	F	R	3	1.3 (20/400)	980	PPV + APC	PPV + ILM peeling + APC	1.2 (20/300)	110	3	3	137	Cataract operation after 2^nd^ surgery in another eye center performed
3	31	F	R	12	0.7 (20/100)	1004	PPV + APC	PPV + APC	0.4 (20/50)	272	SRF till the last follow-up examination	IRF till the last follow-up examination	1	
4	20	F	R	1	0.7 (20/100)	1400	PPV, no ILM peeling	PPV + APC	0.7 (20/100)	273	SRF till the last follow-up examination	IRF till the last follow-up examination	8	
5	27	M	L	Unknown (amblyopia)	1.2 (20/300)	640	PPV + ILM peeling	PPV + APC	0.6 (20/80)	231	SRF till the last follow-up examination	IRF till the last follow-up examination	70	
6	76	F	L	10	0.4 (20/50)	869	PPV + APC	No	0.1 (20/25)	233	13	25	31	Retinal detachment 6 weeks and cataract operation 18 months after surgery
7	25	F	L	5	1.3 (20/400)	904	PPV + APC	No	0.8 (20/125)	344	13	IRF till the last follow-up examination	59	
7	27	F	R	1	0.5 (20/60)	579	PPV + ILM peeling + APC	No	0 (20/20)	186	3	IRF till the last follow-up examination	36	
8	43	F	L	5	0.7 (20/100)	1360	PPV + ILM peeling + APC	PPV + APC	0.8 (20/125)	138	21	21	68	Cataract operation 21 months after 2^nd^ surgery
9	28	F	L	0	0.5 (20/60)	559	PPV + APC	No	0.2 (20/30)	317		IRF till the last follow-up examination	117	Cataract operation 10 months after surgery
10	23	F	R	4	1 (20/200)	915	PPV + APC	No	0.3 (20/40)	137	24	36	48	

*BCVA* best corrected visual acuity, *PPV* pars plana vitrectomy, *ILM* internal limiting membrane, *APC* autologous platelet concentrate, *SRF* subretinal fluid, *IRF* intraretinal fluid, *M* male, *F* female, *R* right, *L* left

A macular detachment was detected in all eyes preoperatively. Furthermore, intraretinal separation (inner or outer schisis, or a combination of both) was also identified in all cases. All patients underwent a 23-gauge three-port PPV. In addition, ILM peeling was performed primarily in four eyes.

The mean duration period of visual loss prior to surgery was 4.7 ± 3.89 months (range 0–12 months).

The foveal thickness decreased significantly from 935.82 ± 248.48 preoperatively to 226.45 ± 76.09 µm at the last follow-up examination (*p* < 0.0001). A gradual postoperative morphological improvement was observed in all cases. A complete resolution of subretinal fluid was observed in eight eyes and in five eyes of them was observed also a complete resolution of intraretinal fluid (in two cases at the same follow-up examination and in three cases in a later follow-up examination). The mean duration of the period up to the final anatomical improvement was 8.6 ± 7.85 months (range 1–25 months). In all cases, no recurrence was observed.

The mean BCVA increased significantly from 0.82 ± 0.33 logMAR (range 0.4–1.3) preoperatively to 0.51 ± 0.36 logMAR (range 0–1.2) at the final follow-up examination (*p* = 0.0022).

The mean follow-up period after the last operation was 65.36 ± 48.81 months (range 1–144 months).

The parameters that can influence the final anatomical and functional outcomes were also investigated in our study. A significant positive correlation was observed between initial and final BCVA (*p* = 0.0101, *r* = 0.73), meaning that the lower the initial BCVA, the poorer the visual prognosis will be. The initial foveal thickness was positively correlated with the final BCVA logMAR, therefore also indicating a poor functional prognosis in patients with excessive ODP-M. However, this correlation did not reach statistical significance (*p* = 0.0951, *r* = 0.53).

No intraoperative complications were observed. Postoperatively, two eyes developed a retinal detachment, which has since been successfully treated with vitrectomy and gas tamponade. Cataract surgery was performed in 5 eyes because of the progression of the lens opacification during the follow-up period.

## Discussion

Our study showed that PPV combined with APC yields a significant improvement of both the visual and the morphological outcomes of patients with ODP-M and it also has a good safety profile. This technique demonstrated successful results not only as a primary but also as a rescue treatment. To the best of our knowledge, this is the longest observation period regarding the use of APC in treatment of ODP-M.

The optic disc pit maculopathy can cause permanent visual loss in eyes with ODP. Without surgical treatment, a spontaneous resolution of the maculopathy was reported in 25% of the cases [19, 20]. Although there are no established guidelines regarding the surgical treatment of the ODM-P, vitrectomy is considered to be the main operative approach. The surgical induction of PVD is regarded as an essential part of the PPV. It is believed that PVD releases the vitreous traction over the ODP, leading to improvement or even resolution of ODP-M [21–23]. Further surgical steps like laser treatment, gas tamponade, ILM peeling, retinal fenestration, or glial tissue removal combined with the PPV were trialed with good clinical results [22, 24–31]. Another approach that has been trialed was to cover and stuff the optic disc pit during vitrectomy. Babu et al. compared two such techniques: inverted ILM flap and autologous scleral plug, with each other and also against PPV with ILM peeling alone [32]. These two techniques were found to be equally effective regarding the anatomical improvement, and both are superior in comparison with ILM peeling alone.

The use of APC has been previously reported as a rescue therapy for failed macular hole surgery [17]. Cullinane et al. investigated the effects of APC and autologous serum on retinal wound healing in an animal model [33]. They reported a greater healing response with APC than with the serum or control group, and they suggested that the presence of a platelet plug may act as a “depot” source of growth factors, thus improving the retinal wound healing. Rosenthal et al. described for the first time the use of APC combined with PPV, with good clinical results in a female patient with persistent ODP-M. They described it as a rescue therapy, to be implemented after a primary treatment with PPV, removal of the posterior hyaloid, and SF6 injection [13]. Todorich et al. also described the use of APC combined with ILM peeling as a rescue therapy, with good results for a female patient with persistent ODP-M, who primarily underwent PPV, peripapillary laser, and gas tamponade [34]. Figueroa et al. reported three cases with good clinical results as well, in which APC combined with ILM peeling in two of the three cases was used as rescue therapy in previously vitrectomized eyes with persistent ODP-M [35]. Nadal et al. reported successful and stable anatomical and functional results in a prospective study of nineteen eyes with ODP-M, each of which also underwent PPV combined with APC, posterior hyaloid peeling, and gas tamponade [36]. They reported the complete resolution of intraretinal schisis-like separation and macular detachment in all patients, without a need for reoperating during the follow-up period.

Previous studies were able to demonstrate the improvement of the ODP-M through surgical induction of PVD alone, therefore eliminating any need for the tractive vitreoretinal components [21–23]. In our study, the use of APC combined with induction of PVD with or without ILM peeling demonstrated successful anatomical results with fovea reattachment in all cases, a significant increase of the final BCVA, and no recurrence during the follow-up. The healing ability of APC on the retinal wound seems to contribute to an improved sealing process of the hypothetical communication between the vitreous cavity and the subretinal space, which reduces the amount of sub- and intraretinal fluid. In our opinion, one of the main advantages of APC injection is that mechanical stress is reduced to a minimum during surgery. In contrast, all stuffing techniques are a more invasive approach as they necessarily include additional mechanical forces on the papilla and peripapillary area, risking further damage to this sensitive region.

Two cases developed retinal detachment postoperatively and have been successfully treated with vitrectomy and gas tamponade. Retinal detachment is a known complication after macular surgery due to iatrogenic retinal breaks, probably during surgical detachment of the posterior vitreous face [37–39].

In two cases (cases 2 and 8), the postoperative foveal thickness was less than 150 µm, not allowing any visual improvement. Retinal atrophy is a known morphological change after resolution of persistent subretinal fluid over a long period [40].

The limitations of this study include its retrospective design and the small number of patients. Additionally, the lack of multiple contributing surgeons narrows the applicability of the outcomes.

In conclusion, APC with PPV can result in successful visual and anatomical outcomes, both as a primary and rescue therapy. Due to the successful results, the less traumatic character, and the good safety profile, this technique represents a promising therapeutic option for ODP-M. Further studies with prospective designs and bigger sample sizes are required to confirm this.

### Supplementary Information

Below is the link to the electronic supplementary material.

Supplementary file1 (MP4 67.4 MB)
